# Prognostic impact of radiotherapy timing in WHO grade 2 and 3 meningiomas utilizing an integrated molecular-morphologic classification

**DOI:** 10.1007/s11060-026-05508-4

**Published:** 2026-03-23

**Authors:** Claire Delbridge, Helen X. Hou, Thomas Hielscher, Benedikt Wiestler, Chiara Negwer, Lena Schenck, Jan Peeken, Christian Diehl, Kai Borm, Sandro Krieg, Kaywan A. Aftahy, Sophia M. Leiss, Friederike Schmidt-Graf, Meike Mitsdörffer, Igor Yakushev, Andreas Von Deimling, Jens Gempt, Bernhard Meyer, Stephanie E. Combs, Felix Sahm, Denise Bernhardt

**Affiliations:** 1 Department of Neuropathology and Pathology, School of Medicine, TU Munich, 81675 München, Germany; 2 Department of Radiation Oncology, Klinikum rechts der Isar, Technical University of Munich (TUM), Ismaninger Straße 22, 81675 Munich, Germany; 3 Department of Biostatistics, German Cancer Research Center (DKFZ), Heidelberg, Germany; 4 Department of Neuroradiology, Klinikum rechts der Isar, TU Munich, 81675 München, Germany; 5 TranslaTUM, TU Munich, 81675 München, Germany; 6 Department of Neurosurgery, Klinikum rechts der Isar, TU Munich, 81675 München, Germany; 7 Department of Neurosurgery, International Neuroscience Institute, Hannover, Germany; 8 Department of Nuclear Medicine, Klinikum rechts der Isar, TU Munich, 81675 München, Germany; 9 Department of Neurosurgery, University Medical Center Hamburg-Eppendorf, 20251 Hamburg, Germany; 10 Institute of Radiation Medicine (IRM), Department of Radiation Sciences (DRS), Ingolstädter Landstraße 1, Neuherberg, Germany; 11 Deutsches Konsortium für Translationale Krebsforschung (DKTK), Partner Site Munich, Munich, Germany; 12 Department of Neuropathology, Institute of Pathology, Ruprecht-Karls-University Heidelberg, Heidelberg, Germany; 13 Clinical Cooperation Unit Neuropathology, German Cancer Research Center (DKFZ), Heidelberg, Germany; 14 Department of Neurosurgery, University Heidelberg, Heidelberg, Germany; 15 Department of Neurology, Klinikum rechts der Isar, TU Munich, 81675 München, Germany

**Keywords:** Meningiomas, Adjuvant radiation therapy, Risk stratification, DNA methylation, Molecular classification

## Abstract

**Purpose:**

Meningiomas (MNGs) occur in different histopathological subtypes. The WHO grading system classifies a subset as grade 2 and 3, indicating a more aggressive course. Recent advances in risk stratification introduces an integrated molecular-morphological score (IntS), offering improved risk prediction over the traditional WHO classification. This study aims to evaluate the prognostic utility of IntS in the context of the timing of adjuvant radiotherapy (RT).

**Methods:**

This retrospective study analyzed 55 patients with histologically diagnosed WHO grade 2 and 3 MNG treated with adjuvant RT. Molecular analyses using Illumina 450k Human BeadChip and Illumina 850k EPIC stratified patients into 3 risk groups (low, intermediate and high) using an integrated model that combines WHO grading, Copy Number Variations (CNVs), and Methylation Families (MF).

**Results:**

After 5 years a local failure-free survival (LFFS) rate of 0% in MF-malignant MNG contrasts with a LFFS rate of 77% and 58% in MF-benign and MF-intermediate MNG. A significant correlation between CNVs and LFFS was also observed in the adjuvant setting. The IntS model revealed distinct 5-year LFFS disparities across different risk categories, underscoring the impact of combined morphological and molecular characteristics on outcome.

**Conclusion:**

The integration of DNA-methylation and CNV-profiles into the IntS unified risk score offer an enhanced prognostic differentiation of MNG patients. This approach shows a promising direction for guiding the optimal timing of adjuvant RT, offering a path toward more tailored treatment strategies for meningiomas.

## Introduction

Meningiomas (MNGs), the most common primary intracranial tumors, have various histological subtypes. Most MNGs are considered benign WHO grade 1 tumors, whereas WHO grade 2 and 3 MNGs together comprise about 20–30%, indicating atypical or anaplastic features associated with a more aggressive clinical course [1, 2]. The role of radiotherapy (RT) in the adjuvant treatment for WHO grade 2 and 3 MNGs is currently debated. According to the European Association of Neuro Oncology (EANO) guidelines, adjuvant RT is recommended for WHO grade 2 MNGs post Simpson IV-V resection and for WHO grade 3 MNGs after radical surgery followed by fractionated radiotherapy [3].

Recent studies show that CNV- and methylation family-based subgrouping improves recurrence risk prediction compared to the current morphology-based WHO classification. An integrated molecular and morphologic risk scoring (IntS) approach combines information from the DNA methylation families (MFs), WHO grading, and the analysis of copy-number changes in chromosomal arms 1p, 6q, and/or 14q, provides superior risk group delineation. The primary aim of this study is to investigate the role of adjuvant RT in the context of CNV and MF based subgrouping in MNGs using the IntS.

## Materials & methods

Molecular analyses were performed at the Department of Neuropathology, University Hospital of Heidelberg, Germany. Fifty-five patients with histologically diagnosed WHO grade 2 and 3 MNG at the Department of Neuropathology who received adjuvant radiotherapy at the Department of Radiation Oncology, Technical University of Munich, Germany, were included. Patients were treated between 2004 and 2021. Only the initial RT and earliest methylation array per patient were analyzed. Target volume definition and progression assessments relied on MRI imaging, with DOTATOC PET/CT increasingly incorporated in recent cases or improved precision.

Illumina 450k Human BeadChip (Illumina, San Diego, CA, USA) and 850k EPIC (Illumina) analyses were used for methylation and copy number analysis. All patients were stratified into 3 risk groups (low, intermediate and high) using the unified three-tiered risk scoring (IntS) classification, combining CNS WHO grade 2 and 3 based on morphological features, CNVs and DNA methylation families (MFs). Detailed descriptions of the materials and methods used have been published previously. The Heidelberg brain tumor classifier v12.5 was used for the DNA methylation-based classification, assigning patients to three MFs comprising six specific methylation classes (MC): MF-benign (36,4%), including MC-benign (ben-1, ben-2, ben-3), MF-intermediate (54.5%), encompassing MC-intermediate (int-A, int-B) and MF-malignant (9.1%), consisting solely of MC-malignant (mal). It should be noted that MC and MF designation are assigned independently by the classifier, based on calibrated confidence scores computed at each level; as a result, MC and MF may not always coincide. The analyses revealed common CNVs, including loss of chromosome arm 1p (56,4%), loss of 6q (0%), and loss of 14q (7,7%) in the patient cohort. The combined data facilitated a comprehensive risk stratification within the IntS framework as described previously by Maas et al [4].

### Statistical methods

Local failure free survival (LFFS) was defined as time from first resection to progression/recurrence or death. Progression/recurrence were defined as new or unequivocal enlargement of residual or recurrent tumor on follow-up MRI. Imaging assessments were performed according to routine institutional practice. The Kaplan-Meier method was used and the differences in survival were assessed using the log-rank test and Cox regression. Missing values were imputed using multiple imputation by chained equations (MICE). Early adjuvant RT was defined as RT administered within 6 months following the initial resection. Late RT was defined as RT received at the time of recurrence or progression. Consequently, this group is expected to represent patients with more aggressive underlying disease, and any comparison with early RT must be interpreted with caution given the inherent indication related bias of retrospective analyses, which cannot be fully mitigated even with Covariate Balance Propensity Score (CBPS) and Inverse Probability of Treatment Weighting (IPTW). All patients included had a follow-up without local failure of at least 6 months (landmark analysis). CBPS analysis accounted for covariate imbalances between the RT treatment groups. IPTW based on propensity scores was used to assess the effect of early RT in Cox regression and adjusted survival curves. P-values < 0.05 were considered statistically significant. Statistical analyses were performed with R using add-on packages mice, CBPS, MatchThem, and adjusted Curves.

### Ethics approval

The study was approved by the ethics committee of the Technical University in Munich, Germany (645/21 S-KK). Informed consent was waived by the ethical committee due to its retrospective design. All examinations and evaluations were performed following institutional guidelines and the Declaration of Helsinki of 1975 in its most recent and updated version.

## Results

### Patient characteristics

Fifty-five patients with a median age of 61 years at the time of RT (range 22–82 years) were available for analysis. The cohort predominantly consisted of patients with atypical meningioma (*n* = 47), followed by clear cell (*n* = 3) and anaplastic MNG (*n* = 5). Accordingly, 50 patients were classified as WHO grade 2 and 5 patients as WHO grade 3. 13 patients underwent subtotal resection and 37 patients underwent complete resection. The resection status was indeterminate for 5 patients. Table 1 lists further patient characteristics. In our cohort a total of 38 patients received early RT as an adjuvant treatment immediately after their initial surgical resection, while the remaining 17 patients underwent late RT, initiated more than 6 months after initial surgery as a salvage therapy following disease progression or re-resection. All patients were treated with fractionated photon RT. The majority of patients (96.4%) received normofractionated RT, with only a small number of patients in the late RT group receiving FSRT (5.9%) and SRS (5.9%). Table 2 presents the detailed characteristics of RT and the timing of treatment for patients within the cohort.

**Table 1:** Demographic, clinical and molecular characteristics of patients with meningioma

	Overall (*N* = 55)
**Morphological Subtype**	
Anaplastic Meningioma	5 (9.1%)
Atypical Meningioma	47 (85.5%)
Clear Cell Meningioma	3 (5.5%)
**WHO Grading**	
2	50 (90,9%)
3	5 (9,1%)
**Age at Resection**	
Median (Min, Max)	61.0 [22.1, 82.1]
**Karnofsky Performance Status (KPS)**	
Median (Min, Max)	90.0 [50.0, 100]
Missing	2 (3.6%)
**Resection status**	
Simpson I	14 (25.5%)
Simpson II	17 (30.9%)
Simpson III	6 (10.9%)
Simpson IV	13 (23.6%)
Missing	5 (9.1%)
Methylation Family (MF)*	
ben	20 (36.4%)
int	30 (54.5%)
mal	5 (9.1%)
Methylation Class (MC)*	
ben.1	10 (18.2%)
ben.2	4 (7.3%)
ben.3	2 (3.6%)
int.A	29 (52.7%)
int.B	3 (5.5%)
mal	7 (12.7%)
**Chromosomal Copy Number Alterations**	
loss of 1p	31 (56.4%)
loss of 6q	0 (0%)
loss of 14q	4 (7.7%)
**Copy Number Variations Group**	
0	24 (43.6%)
1	27 (49.1%)
2	4 (7.3%)
**IntS**	
low	14 (25.5%)
int	35 (63.6%)
high	6 (10.9%)

**Table 2:** Characteristics of RT and Timing – Early vs. Late RT Intervention Characteristics in Meningioma Patients

	Overall (*N* = 55)	Late RT (*N* = 17)	Early RT (*N* = 38)
**Time Primary Diagnosis to RT (Months)**			
Mean (SD)	21.5 (43.0)	44.9 (52.6)	11.0 (33.7)
Median (Min, Max)	4.57 [1.09, 222]	34.7 [4.11, 222]	3.36 [1.09, 201]
**Time Resection to RT (Months)**			
Mean (SD)	16.1 (34.6)	45.3 (52.2)	3.04 (1.33)
Median (Min, Max)	4.18 [0.921, 222]	33.5 [6.94, 222]	2.89 [0.921, 5.49]
**RT Technique**			
SRS	1 (1.8%)	1 (5.9%)	0 (0%)
FSRT	1 (1.8%)	1 (5.9%)	0 (0%)
Normofractionated RT	53 (96.4%)	15 (88.2%)	38 (100%)
**RT Dose (Gy)**			
Mean (SD)	54.2 (11.9)	52.5 (10.8)	55.0 (12.4)
Median (Min, Max)	59.4 [12.6, 60.0]	54.0 [17.0, 60.0]	59.4 [12.6, 60.0]

Median LFFS was 71 months for WHO Grade 2 and 30 months for WHO Grade 3 MNGs. For OS the median follow-up time was 61 months with a 5‐year survival rate of 88%. The classification of MNGs based on their methylation profiles into intermediate and malignant tumors aligns with the histologically assigned WHO grading, demonstrating significant prognostic implications for the LFFS. At the 5-year mark, the local failure-free survival (LFFS) rate was 0% in patients in the malignant group, in stark contrast to LFFS rates of 77% and 58% observed in patients with benign and intermediate MNGs.

**Fig. 1:**
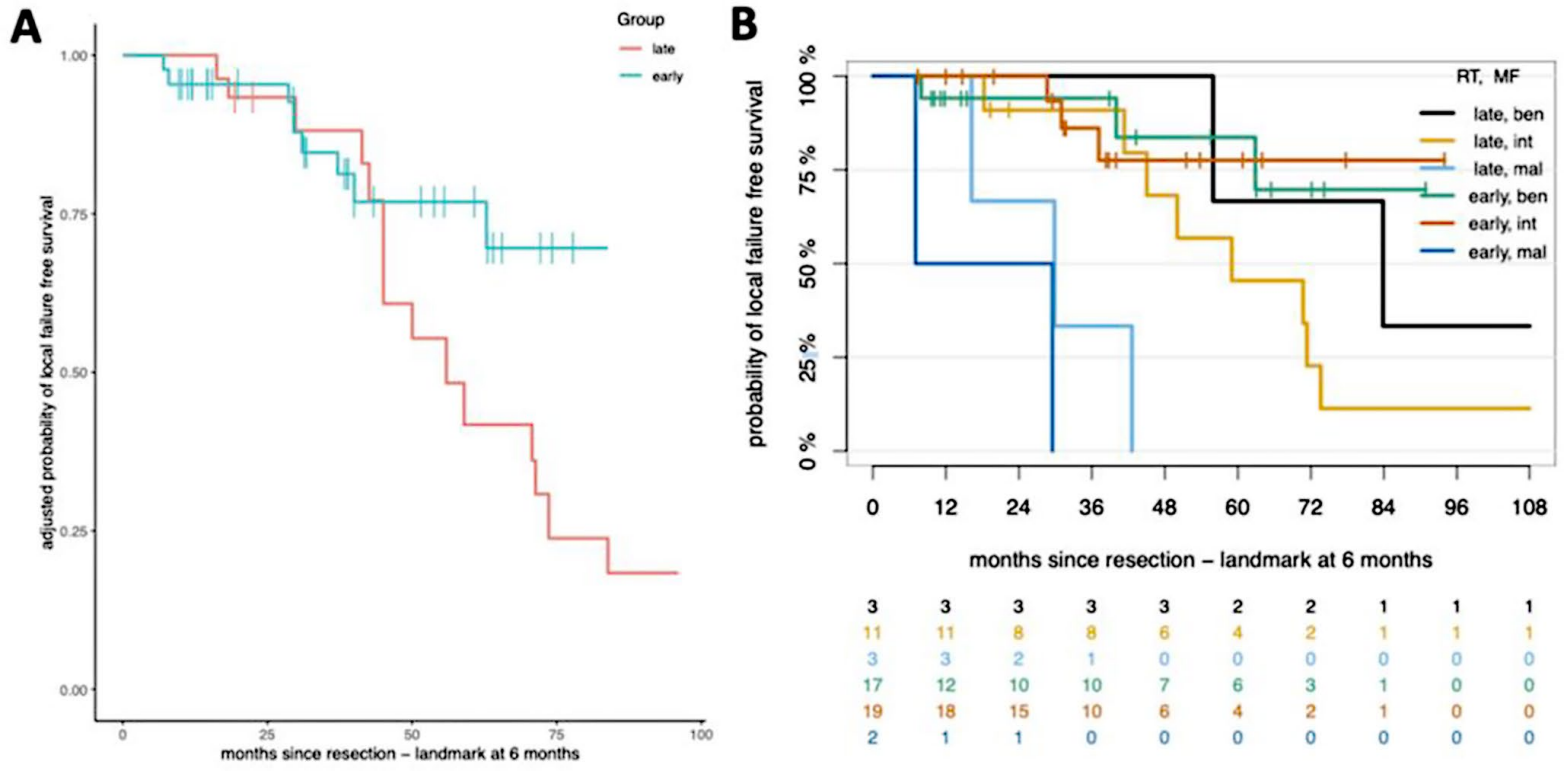
Kaplan-Meier survival plots. (**A**) Adjusted probability of LFFS for early versus late radiotherapy in meningioma patients, utilizing an Inverse Probability of Treatment Weighting (1PTW) approach. (**B**) Probability of LFFS based on Methylation Family status. P value, log-rank test

The effect of adjuvant RT timing on LFFS was analyzed and adjusted survival curves via an IPTW approach revealed a distinct but non-significant difference between early and late RT groups (Fig. 1A, IPTW adjusted Hazard ratio 0.42 95% CI 0.16–1.10, *p* = 0.07). Patients receiving early RT demonstrated a 75% probability of LFFS, compared to a lower percentage (40%) in the late RT cohort at 5 years. In the subgroup analysis the intermediate MNG group exhibited a potential benefit from early postoperative RT (*N* = 19). The early RT group had not reached median LFFS within the observation period, while the late irradiated group (*N* = 11) reached a median LFFS at 59 months, although the subgroup sizes in this analysis were relatively small and did not reach statistical significance (IPTW adjusted HR = 0.23, 95% CI 0.05–1.14, *p* = 0.07). No significant LFFS differences were found when comparing early and late RT across the benign and malignant MNG groups. When comparing early and late RT, there were no statistically significant differences between the benign and malignant MNG groups. Despite this, the potential LFFS advantage seen with early adjuvant RT in the intermediate MNGs (MF int) (*N* = 19), highlights the need for further investigation in larger cohorts (Fig. 1B).

**Fig. 2:**
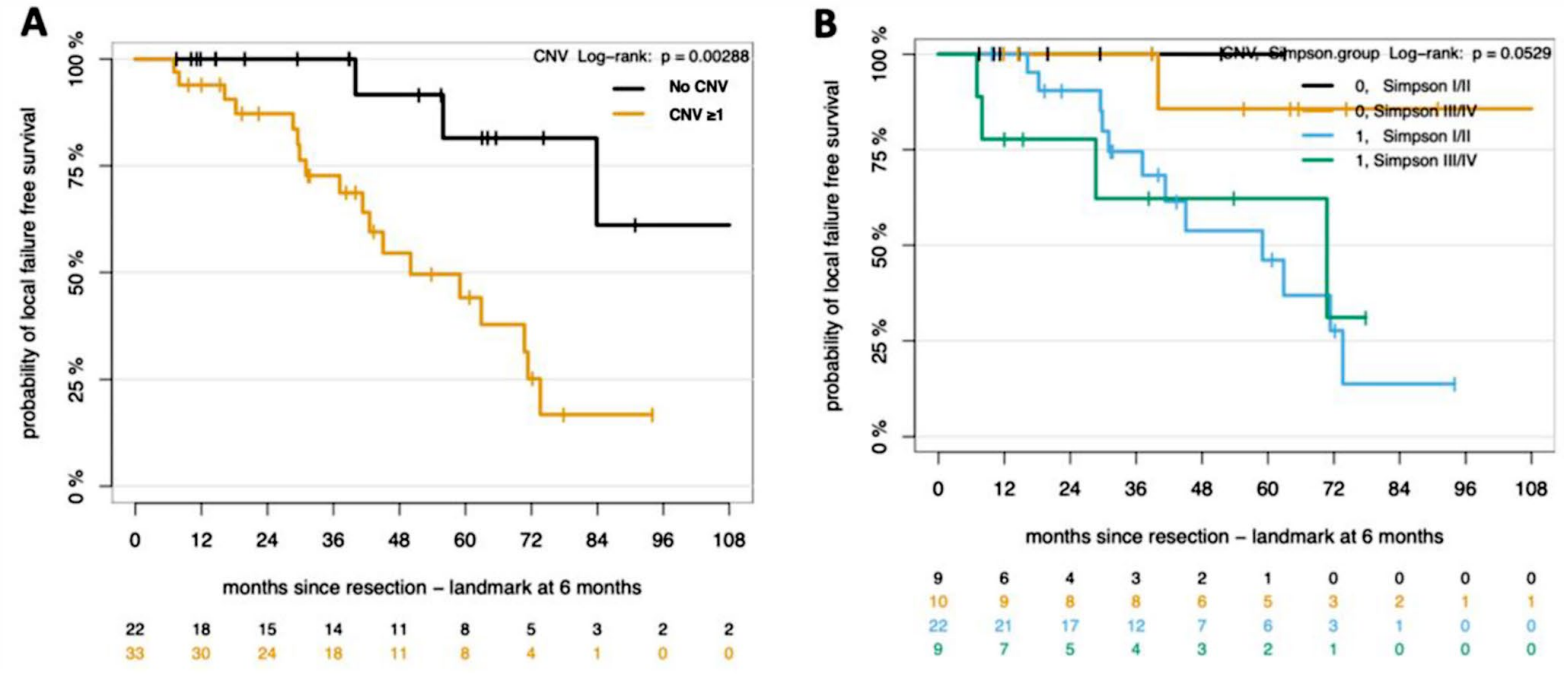
Kaplan-Meier survival plot. Assessing LFFS by CNV Status (**A**) and surgical resection status according to Simpson Grading (**B**). P value, log- rank test

**Fig. 3:**
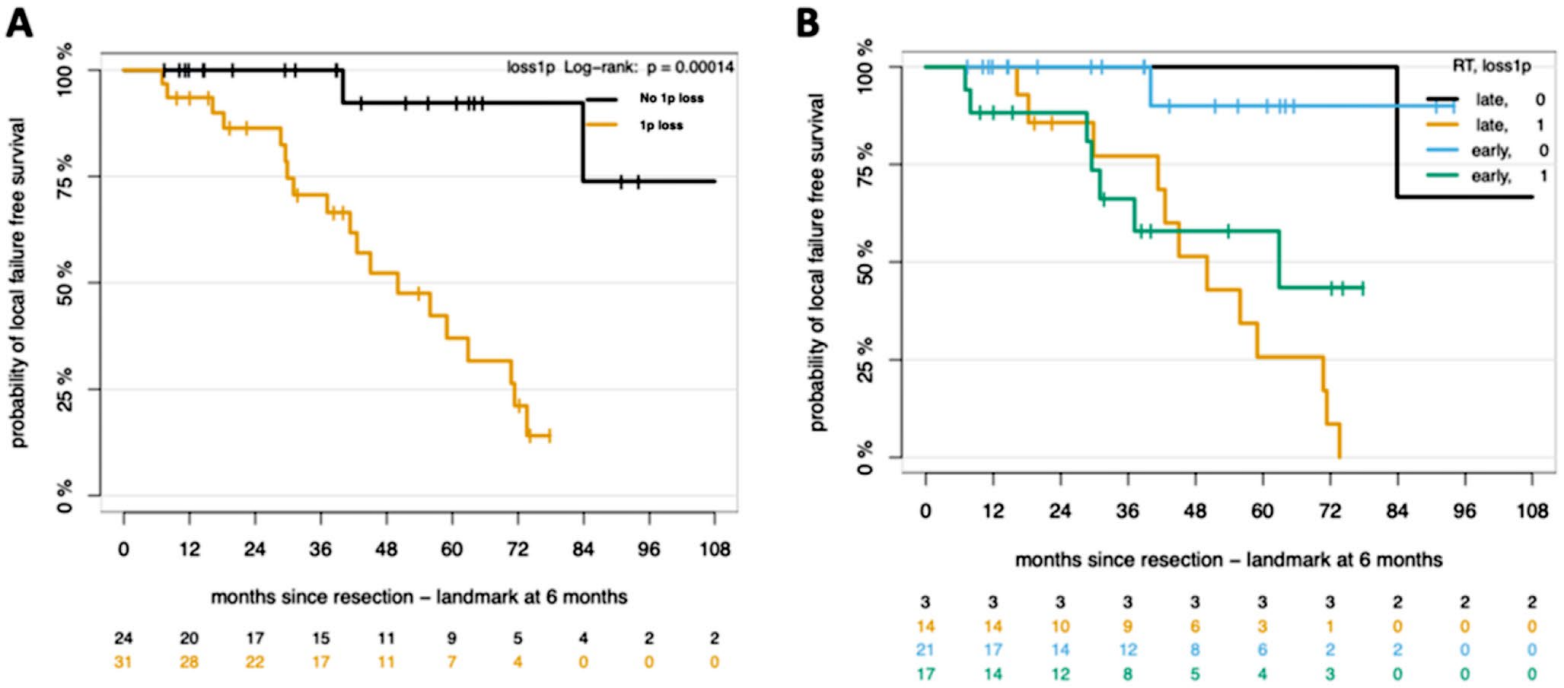
Kaplan-Meier survival plots. (**A**) Probability of LFFS in patients with and without CNV lp loss. (**B**) Survival analysis illustrating the combined effect of RT timing (early vs. late) and CNV lp status on LFFS. P values, log-rank test

Copy number variations (CNVs) emerge as highly predictive markers for the outcomes of WHO Grade 2 and 3 MNGs in the adjuvant setting. The CNV subgroup analysis reveals a statistically significant (*p* = 0.0029) difference in LFFS between patients with and without CNVs (Fig. 2A). Patients without CNVs demonstrated a higher probability of LFFS after 5 years (80%), in contrast to those with CNVs, who exhibited a markedly lower LFFS (44%). We conducted further analysis focusing on the loss of chromosomal arm 1p, due to its notable high occurrence in 56% of our patient cohort. This genomic alteration has been proven in existing literature to be associated with higher risk of progression or recurrence [4]. Our results further support the prognostic significance of 1p loss. Patients exhibiting 1p loss displayed a markedly reduced 5-year LFFS of 36.9%, compared to 92.3% in those without 1p loss (Fig. 3A). The statistical significance of these differences was confirmed (*p* = 0.0001). However, when examining the effect of 1p loss and timing of adjuvant RT – early versus late – no difference in RT effect between 1p groups on LFFS was observed (*p* = 0.78) (Fig. 3B).

Subgroup analysis based on Simpson Grading yielded no statistically significant differences in LFFS outcomes between different Simpson Grade categories when considering methylation groups and CNVs (*p* = 0.053) (Fig. 2B). These findings suggest that the extent of tumour resection, as reflected by Simpson Grading, may not be as critical to the LFFS outcomes as the molecular characteristics of the tumor itself, particularly in patients who have received RT.

**Fig. 4:**
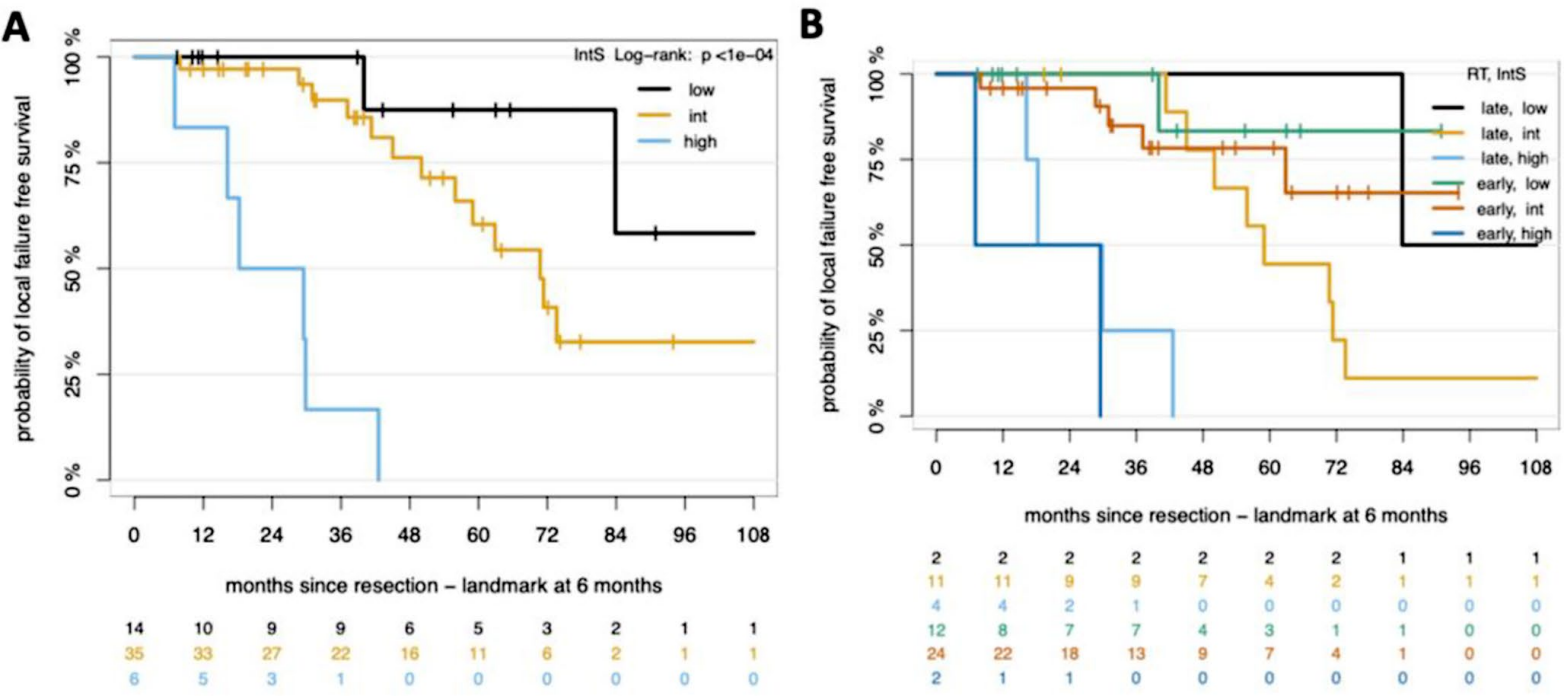
Kaplan-Meier survival plots. (**A**) Probability of LFFS in patients by the integrated molecular and morphologic risk scoring IntS. (**B**) Survival analysis illustrating the combined effect of RT timing (early vs. late) and each IntS category on LFFS. P values, log-rank test

The risk stratification based on integrated molecular-morphologic criteria (IntS) yielded notable differences in 5-year LFFS. The IntS-low-risk group (*n* = 14) demonstrated a 5-year LFFS of 87,5%, while the IntS-intermediate group (*n* = 35) displayed a LFFS of 60%, and the IntS-high-risk group (*n* = 6) had a LFFS of 0%. These differences in LFFS were statistically significant (*p* < 0.0001) (Fig. 4A). In the analysis of LFFS among IntS risk categories in relation to the timing of RT (early vs. late), the IntS-low risk group demonstrated consistently high LFFS, with no substantial difference between early and late RT. In contrast, the IntS-high risk group presented with low LFFS regardless of the timing of RT. In the IntS-intermediate MNG group, a trend towards longer LFFS was observed in patients receiving early adjuvant RT compared to late adjuvant RT (Fig. 4B). The adjusted median LFFS was not reached compared to 59 months for the late irradiated group and this difference did not reach reach statistical significance (IPTW adj HR = 0.31, 95% CI 0.09–1.12, *p* = 0.07). 

## Discussion

Prior to the 2021 CNS WHO classification, grading and risk assessment for MNGs relied exclusively on morphological attributes. The 2021 classification (CNS5) has introduced TERT promoter mutation and homozygous deletion of CDKN2A/B as first molecular markers, but these occur in only a small fraction of MNGs and do not further stratify low and intermediate risk cases. DNA methylation-based brain tumor classification has emerged as a valuable diagnostic tool, with significant implications for predicting tumor recurrence. DNA methylation profiling in MNG identified six clinically relevant classes grouped into three overarching methylation class families [5]. These were closely linked to characteristic mutational, cytogenetic, and gene expression patterns. Further studies have supported the role of methylation in meningioma subgrouping [6–8]. Subsequently, the integrated molecular and morphologic risk scoring (IntS) [4] that combines DNA methylation class, CNS WHO grading, and CNVs in chromosomal arms 1p, 6q, and/or 14q was developed, and has improved diagnostic accuracy over the conventional CNS WHO grading system. Our study uses this integrated molecular-morphologic classification system [4], this approach is related to, but distinct from, other classification systems, such as the methylation-based schemes from BWH and UCSF, each highlighting unique meningioma subtypes and risk features [6, 9]. Gene expression-based methods, such as Chen et al.’s, further refine therapeutic targeting by exploring additional molecular pathways [10].

Most previous studies on DNA methylation-based tumor scores did not account for adjuvant RT or its timing. In our patient group, RT data was available for all patients, and the integration of molecular and morphological criteria has revealed differences in 5-year LFFS between risk groups. Notably, early adjuvant RT appeared to benefit a subgroup of patients with intermediate-grade (MF int) MNGs (*N* = 19); however, given the small sample size and wide confidence intervals, this observation should be interpreted with caution. While no significant differences in LFFS were found between early and late RT, the observed directional trend suggesting a benefit of early RT in molecularly selected subgroups is encouraging and supports the hypothesis that RT timing may be a clinically meaningful variable. These findings are exploratory and will require prospective validation in larger cohorts.

Several retrospective series have previously examined the impact of postoperative RT timing in atypical meningioma and reported that early adjuvant RT was associated with improved LC [11, 12]. Our findings are consistent with the notion that earlier RT may benefit at least a subset of patients and extent these findings by incorporating molecular risk stratification to identify those at highest risk who may derive the greatest benefit from early adjuvant RT.

Ehret et al. (2024) showed that DNA methylation-based integrated risk classification in WHO Grade 2 MNGs are prognostic for local control, but limited adjuvant RT use (15/100 patients) restricts conclusions about its impact, especially for intermediate and high-risk patients [13]. Deng et al. explored the recurrence probability following RT in 44 patients with CNS WHO Grade 2 MNGs, using an integrated molecular-morphologic classification to stratify patients into low, intermediate, and high molecular risk groups [14]. The study identified significant differences in 3-year local progression-free survival (lPFS): 100% in low-risk, 89.5% in intermediate-risk, and 75.5% in high-risk groups, demonstrating the integrated model’s robustness in predicting recurrence risk post-RT [14]. Notably, 87.5% of recurrences occurred within the RT planning target volume (PTV) or resection cavity, while distant out-of-field recurrences were more common in the high-risk group. A significant limitation in Deng et al. is the uniformity of RT timing, with all patients receiving RT immediately post-surgery [14]. This lack of timing variation restricts the study’s ability to evaluate whether early or delayed RT might yield different outcomes, particularly for high-risk patients. Timing variation, as shown in other studies, could provide insights into optimal RT initiation for various molecular subgroups and potentially improve treatment personalization. This homogeneous RT approach limits the findings, especially in high-risk cases, where delayed RT might impact recurrence patterns and progression. A large, multi-institutional retrospective study by Wang et al., analyzed 2,035 MNG cases, of which 638 are WHO grade 2 and 199 are WHO grade 3 [15]. The study evaluates the prognostic impact of surgical resection extent and molecular classification on progression-free survival (PFS) and overall survival (OS) across molecularly defined subtypes: Immunogenic, NF2-wild-type, Hypermetabolic, and Proliferative. The findings showed that molecular subtype predicts recurrence risk, with Proliferative tumors being most resistant to RT; results were robust due to propensity score matching. However, detailed RT timing was not assessed, limiting interpretation for adjuvant RT optimization [15]. Maas et al. integrates findings from the EORTC 22042–26042 study, particularly for WHO grade 2 meningiomas following gross total resection and adjuvant RT, reinforcing the utility of molecular markers such as DNA methylation classes and chromosomal 1p loss to predict recurrence [16]. Maas et al. emphasizes a broader analysis across grade 2 and 3 meningiomas but does not explore the implications of adjuvant RT timing and dosage effects in detail.

The NRG Oncology/RTOG 0539 trial investigated outcomes of patients with clinicopathologically classified intermediate-risk meningiomas [17]. In a separate clinical outcomes report from the same NRG/RTOG trial, clinicopathologically classified high-risk meningiomas were evaluated. These high-risk cases were characterized by either new or recurrent WHO grade 3 tumors or recurrent WHO grade 2 meningiomas of any resection extent, or newly diagnosed WHO grade 2 tumors following subtotal resection. The 3-year PFS was 58.8%, local control reached 68.9%, and the 3-year OS was 78.6%. In the European EORTC 22042–26042 phase II study, 56 patients with newly diagnosed WHO grade 2 MNGs who had undergone gross total resection and received fractionated RT were evaluated [18]. A total radiation dose of 60 Gy was delivered in 2.0 Gy per fraction. The results demonstrated a 3-year PFS of 88.7%, an OS of 98%, and a local failure rate of 14.3%. The findings from both the US and European trials collectively suggest the potential benefits of fractionated RT for patients with clinicopathologically classified intermediate and high-risk MNGs. This finding is also supported by a recent review [19]. Data from 30 studies involving 2904 patients (adjuvant RT: *n* = 737; observation: *n* = 2167) were included. Significant reduction of local recurrence rate was seen in the adjuvant RT cohort compared to the observation cohort (OR 0.50; 95% CI 0.36–0.68; *p* < 0.0001). However, it remains unclear whether early adjuvant RT reduces the risk of tumor recurrence following gross total resection of WHO grade 2 MNGS and is superior to a wait-and watch approach. The answers to these critical questions may result from the outcome of two ongoing prospective controlled phase III trials. These trials involve one group receiving adjuvant RT and the other undergoing observation following surgical resection of atypical MNGs. The recently concluded ROAM/EORTC 1308 trial, and the ongoing NRG-BN003 trial (ClinicalTrials.gov Identifier: NCT03180268), are expected to shed light on this contentious issue. Notably, these trials do not incorporate advanced molecular profiling.

The subgroup analysis of CNVs demonstrated a notable impact on LFFS. In particular, loss of 1p was prevalent in our patient cohort and was associated with a marked decrease in LFFS, confirming its role as a negative prognostic marker. However, 1p loss did not influence LFFS based on RT timing, suggesting its presence alone does not determine the optimal timing of RT. In our cohort CNV status further stratified by Simpson grading showed a noteworthy trend towards an improved LFFS (*p* = 0.053). Large registry studies underscore the importance of both extend of resection and adjuvant RT. A National Cancer Data Base (NCDB) analysis of over 5452 atypical meningiomas that gross total resection (GTR) and timely adjuvant RT were associated with improved OS, with the strongest benefit when RT was initiated within 12 weeks after GTR or between 12 and 6 months after subtotal resection (STR) [20]. The PERNS multicenter cohort of 1452 WHO grade 2 MNGs, similarly reported a consistent reduction in recurrence risk with adjuvant fractionated after STR, while the effect after GTR was less robust and appeared highly dependent on patient selection and institutional practice [21]. Taken together, these studies and our results suggest that Simpson grade, RT timing, and molecular features each contribute to outcome, underscoring the importance of careful patient selection for early adjuvant RT.

The differential impact of IntS on 5-year LFFS outcomes suggests a potential utility of IntS in guiding clinical decision-making. In the IntS low-risk group, the 5- year LFFS of 87,5% aligns with previous reports suggesting more favorable outcomes. Conversely, the IntS high-risk groups 5-year LFFS of 0%, reflects an aggressive disease course that remains refractory to current therapeutic strategies in this subgroup. In the IntS-intermediate group, the comparison of early versus late RT revealed a directional trend. Patients receiving early RT demonstrated a 72 months LFFS of 70%, numerically higher than the 20% observed in their counterparts who received late RT, however this difference did not reach statistical significance (*p* = 0.07). The divergence in LFFS, commencing at around 10 months after resection, may warrant further investigation regarding the potential role of RT in IntS intermediate-risk patients.

While our study provides valuable insights, certain limitations must be acknowledged. The small sample size, retrospective nature and variability in treatment modalities may limit the generalizability of our findings. The comparison between early and late RT is subject to confounding by indication, as the late RT group comprises patients who received RT at recurrence or progression, reflecting an inherently more aggressive disease course that cannot be fully adjusted for in a retrospective analysis. Additionally, defining LFFS from the time of first resection inherently disadvantages the late RT group, as progression is a structural prerequisite for receiving delayed RT, a bias that our landmark analysis only partially mitigates. Data on TERT promoter mutation and homozygous deletion of CDKN2A/B were not available for our cohort. As these markers are now recognized adverse prognostic factors and grade 3 defining molecular criteria for meningioma in the 2021 WHO CNS5 classification [22], their absence limits the completeness of molecular risk stratification in our dataset and may have influenced IntS group assignments. Nonetheless, our cohort reflect real-world clinical data, capturing the diversity and complexity of treatment practices in routine clinical settings.

In conclusion, the molecular characterization of MNGs is emerging as an important tool with promising prospects for enhancing precision and therapeutic efficacy in RT. Our data suggest that methylation profiles have significant prognostic implications for LFFS. A directional trend was observed suggesting that intermediate MF profiles may identify patients who could potentially benefit from early RT. This observation holds clinical weight and calls for further validation in larger cohorts. Furthermore, CNVs have shown their potential as predictive markers for the outcomes of WHO Grade 2 and 3 meningiomas in the adjuvant setting. Our findings support the integration of methylation and CNV profiling as a component in diagnostics and treatment planning for MNGs. The IntS offers a promising approach to prognostic differentiation of MNG and could serve as a stratification tool in future prospective trials to personalize and optimize the postoperative approach to higher grade MNG.

## Data Availability

The data that support the findings of this study are not openly available due to reasons of sensitivity and are available from the corresponding author upon reasonable request.
